# Nutrient availability and acid erosion determine the early colonization of limestone by lithobiontic microorganisms

**DOI:** 10.3389/fmicb.2023.1194871

**Published:** 2023-06-09

**Authors:** Jin Chen, Qing Zhao, Fangbing Li, Xiangwei Zhao, Yang Wang, Limin Zhang, Jinan Liu, Lingbin Yan, Lifei Yu

**Affiliations:** ^1^Key Laboratory of Plant Resources Conservation and Germplasm Innovation in Mountainous Region (Ministry of Education), College of Life Sciences and Institute of Agro-Bioengineering, Guizhou University, Guiyang, Guizhou, China; ^2^School of Mathematical Sciences, Guizhou Normal University, Guiyang, Guizhou, China; ^3^Institute of Guizhou Mountain Resources, Guizhou Academy of Sciences, Guiyang, Guizhou, China; ^4^Garden Greening Center of Logistics Management Office, Guizhou University, Guiyang, Guizhou, China

**Keywords:** biodeterioration, bioreceptivity, lithobiontic microorganism, corrosion, limestone

## Abstract

**Introduction:**

Microorganisms, including the pioneer microorganisms that play a role in the early colonization of rock, are extremely important biological factors in rock deterioration. The interaction of microorganisms with limestone leads to biodeterioration, accelerates soil formation, and plays an important role in the restoration of degraded ecosystems that cannot be ignored. However, the process of microbial colonization of sterile limestone in the early stages of ecological succession is unclear, as are the factors that affect the colonization. Acid erosion (both organic and inorganic), nutrient availability, and water availability are thought to be key factors affecting the colonization of lithobiontic microorganisms.

**Methods:**

In this study, organic acid (Oa), inorganic acid (Ia), inorganic acid + nutrient solution (Ia + Nut), nutrient solution (Nut), and rain shade (RS) treatments were applied to sterilized limestone, and the interaction between microorganisms and limestone was investigated using high-throughput sequencing techniques to assess the microorganisms on the limestone after 60 days of natural placement.

**Results:**

The results were as follows: (1) The abundance of fungi was higher than that of bacteria in the early colonization of limestone, and the dominant bacterial phyla were Proteobacteria, Bacteroidota, and Actinobacteriota, while the dominant fungal phyla were Ascomycota, Basidiomycota, and Chytridiomycota. (2) Acid erosion and nutrient availability shaped different microbial communities in different ways, with bacteria being more sensitive to the environmental stresses than fungi, and the higher the acidity (Ia and Oa)/nutrient concentration, the greater the differences in microbial communities compared to the control (based on principal coordinate analysis). (3) Fungal communities were highly resistant to environmental stress and competitive, while bacterial communities were highly resilient to environmental stress and stable.

**Discussion:**

In conclusion, our results indicate that limestone exhibits high bioreceptivity and can be rapidly colonized by microorganisms within 60 days in its natural environment, and both nutrient availability and acid erosion of limestone are important determinants of early microbial colonization.

## 1. Introduction

Microbial colonization interferes with the integrity and esthetics of rock minerals (Trovão et al., 2019). Worldwide, most lithic artifacts have suffered irreversible biodegradation (Abdel Ghany et al., 2019; Trovão et al., 2019; Gambino et al., 2021; Zhang et al., 2021), for example, the Angkor sandstone monuments (Liu et al., 2018), the limestone walls of the old cathedral of Coimbra (Trovão et al., 2019), the Chaalis abbey (Mihajlovski et al., 2017), and the Feilaifeng limestone statue (Li et al., 2018). Southwest China is a typical carbonate area (Chen et al., 2022), where politics, economics, and culture are all linked to carbonate rocks. For example, policies in Southwest China are linked to the ecological restoration of areas exhibiting karstic desertification, and most building materials are limestone. The study of the biodeterioration of limestone is therefore of great value. Acids (both organic and inorganic) produced by colonizing biota are known key factors that lead to rock biodegradation (Zhang et al., 2019). Stone relic conservation science aims to slow or even eradicate the biodegradation of lithic relics caused by microorganisms and preserve their integrity. In contrast, according to ecological succession theory, acceleration of the biodeterioration of stones leads to the formation of relatively stable biological communities, promotes biomineralization, and accelerates soil formation. The clarification of the ecological succession process of colonizers and their interactions with the stone matrix is exceptionally important both for the conservation of lithic artifacts and for the promotion of soil formation. Microbial biodeterioration involves a series of processes, including biofilm formation, discoloration, salinization, mechanical damage, permeation, and organic matter production (Scheerer et al., 2009). The essence of microbial biodeterioration is the action of hydrogen ions from acidic corrosives produced by lithobiontic microorganisms on the rock matrix, resulting in dissolution, complexation, and chelation (Moroni and Pitzurra, 2008; Gadd, 2017a,b; Li et al., 2018). The acids produced are mainly organic acids such as oxalic and citric acid (Gadd, 1999; de Oliveira Mendes et al., 2020) and inorganic acids, such as HNO_2_, HNO_3_, H_2_SO_3_, and H_2_SO_4_ (Warscheid et al., 1991; Warscheid and Braams, 2000; Moroni and Pitzurra, 2008). The organisms involved in rock biodeterioration mainly include bacteria, Cyanobacteria, fungi, algae, lichens, and mosses (Scheerer et al., 2009; Pinheiro et al., 2019; Zhang et al., 2019). In recent years, the study of lithobiontic microorganisms has made great progress, from determining the role of single microbial species (Gerrits et al., 2021) to determining the role of multiple microbial species (Crispim and Gaylarde, 2005; Trovão et al., 2020) on rocks, and the study of rock biodeterioration is flourishing. Various organisms on rocks, such as fungi, algae, and lichens, have been extensively reported on, but bacteria and archaea have been relatively less reported on (Pinheiro et al., 2019). Regarding fungi, most studies have focused on the biodegradation of limestone caused by fungal strains that can be isolated and cultured (Trovão et al., 2020, 2021), while relatively little attention has been paid to other fungal taxa that colonize limestone under natural conditions.

Because of the poor availability of water and organic matter on rocks, pioneer microorganisms colonizing rocks generally have the ability to utilize small amounts of water, inorganic matter, and airborne organic matter (Villa et al., 2016). In addition, rocks provide little shelter for microorganisms other than the pores and cracks on the rocks, so lithobiontic microorganisms are often exposed to conditions of drastic temperature changes and strong UV light (Walker and Pace, 2007). In response to these conditions, lithobiontic microbes often form specific biofilms based on their nutrient and growth requirements to increase their adaptation to extreme environments (Gorbushina, 2007). Compared to microorganisms in other environments, lithobiontic microorganisms are characterized by (1) low taxonomic diversity but high synergistic and metabolic activity (Villa et al., 2015); (2) phylogenetic similarity and high specificity worldwide (Gorbushina and Broughton, 2009); and (3) pigments, exopolymeric substances, and efficient DNA repair systems that allow survival on the rock (Gómez-Silva, 2018). Rock surface pH, porosity, permeability, mineral composition, texture, geometry, shading, and timing of colonization affect the composition and structure of lithobiontic microbial communities (Miller et al., 2012; Brewer and Fierer, 2018; Liu et al., 2018, 2020; Abdel Ghany et al., 2019; Chen et al., 2022). A decrease in rock surface pH is generally regarded as more serious biodeterioration (Pinheiro et al., 2019). In addition, air is an important factor influencing the structure and composition of lithobiontic microbial communities, especially as air near cities contains organic pollutants that can be a source of energy for microorganisms (Mitchell and Gu, 2000; Villa et al., 2016).

Limestone is one of the rock types that are more susceptible to biodeterioration. Softness, brightness, and easy sculptability increase the bioreceptivity of limestone (Miller et al., 2012; Pinheiro et al., 2019; Chen et al., 2022). The inorganic compounds in limestone are good substrates for the growth of various microorganisms, with microorganisms obtaining the required elements by secreting organic acids (Warscheid et al., 1991). For example, *Nitrosomonas* spp. can secrete nitric acid and *Thiobacillus* spp. can secrete sulfuric acid, thereby obtaining the necessary chemoenergetic nutrients (Warscheid and Braams, 2000). Our previous study reported on the microbial taxa (and their functional genetic variations) on carbonate rock under natural conditions with various weathering times (Chen et al., 2022). However, it is not clear which microorganisms take the lead in colonizing limestone under natural conditions, or whether acid erosion or nutrient availability promote microbial colonization. Therefore, to understand the effects of acid erosion (organic and inorganic acids), nutrient availability, and rainfall on microbial colonization, we set up a total of five treatments: organic acid (Oa), inorganic acid (Ia), inorganic acid + nutrient solution (Ia + Nut), nutrient solution (Nut), and rain shade (RS). We focused on the following questions: what are the early colonizing microbial species on limestone surfaces in the subtropical climate zone? What are the ecological strategies of bacterial and fungal communities during colonization of limestone surfaces? What factors influence the colonization of limestone surfaces by microorganisms? To clarify these questions, we selected sterilized limestone sand-sized grains as the study material and applied different treatments to investigate the colonization patterns of various microbes. Our study provides new insights into the potential conservation of limestone artifacts and the soil-forming role of limestone in karst areas.

## 2. Materials and methods

### 2.1. Experimental design and sample processing

We chose limestone, which is commonly found in southwest China, as the study material. The limestone was purchased from a specialized stone factory and was uniformly processed to a particle size that could pass through a 3-mm but not 1.5-mm sieve. The limestone samples were sterilized in a sterilizer at 180°C for 2 h and cooled. Next, 300 g were placed in a 100 mm × 95 mm × 55 mm plastic grid (Supplementary Figure S1H). Before adding the samples, a sterilized piece of gauze with an approximate pore size of 1 mm was placed in each compartment of the grid to prevent leakage of the added samples. In addition, an *in situ* weather station (Supplementary Figure S1B) was installed to observe the meteorological elements such as temperature, humidity, atmospheric pressure, and rainfall at the experimental site (26°25′42.65″, 106°39′59.65″), and readings were taken every 10 min. The experiment ran from January 15 to March 15, 2022.

To explore the relationship between limestone dissolution and microbial colonization, we set up six groups, i.e., addition of nutrient solution (Nut; Supplementary Figure S1G), addition of organic acid (Oa; Supplementary Figure S1D), addition of inorganic acid (Ia; Supplementary Figure S1E), addition of inorganic acid and nutrient solution (Ia + Nut; Supplementary Figure S1F), control (CK; Supplementary Figure S1C), and rain shade (RS; Supplementary Figure S1C) groups. Hoagland’s solution is a complex nutrient solution containing large amounts of macronutrients and micronutrients required by a variety of organisms. Therefore, for the Nut group, a concentration gradient was set up involving five nutrient concentrations (40 mL each; Supplementary Table S1), i.e., 5, 10, 15, 20, and 25 mL Hoagland’s solution mixed with water (for example, 5 mL Hoagland’s solution in 35 mL water, and so on). The Nut concentrations and ratios were based on previous descriptions (Rajan et al., 2019). We selected oxalic acid, which is commonly found in rocks undergoing biodeterioration, as the corrosive organic acid for this experiment. For the Oa group, we set up a concentration gradient involving five oxalic acid concentrations (40 mL each), i.e., 0.1, 0.2, 0.4, 0.8, and 1.6 mmol/L. The Oa concentrations were based on the concentrations of 0.3–0.7 mmol/L in rocks undergoing biodeterioration reported by Sheng et al. (1997). We selected hydrochloric acid, which is often used as the dissolution acid for carbonate-related experiments (Sun et al., 2010), as the strong dissolution acid for this experiment. For the Ia group, we set up a concentration gradient involving five hydrochloric acid concentrations (40 mL each), i.e., 0.1, 0.2, 0.4, 0.8, and 1.6 mol/L. For the Ia + Nut group, we set up the same concentration gradient of hydrochloric acid as in the Ia group, and after the reaction was completed (after 24 h), we added 15 mL Hoagland’s solution diluted to 40 mL with water. For the Nut, Oa, and Ia groups, after 24 h, 40 mL sterile water was added to give a final volume of 80 mL. For the CK and RS groups, 80 mL sterile water was used. After each rainfall event, we added an equal amount of sterile water to the RS group based on the amount of rainfall recorded by the weather station. We conducted three replicates of each treatment to give a total of 66 samples.

### 2.2. Sampling and assessment of limestone samples

It has been found that fungi grow on modern limestone surfaces after 60 days of infection (Abdel Ghany et al., 2019). Therefore, after the limestone samples had been left outdoors for 60 days, we scooped them out with a sterile steel spoon and placed them in labeled plastic bags. For high-throughput sequencing, to obtain microorganisms samples for DNA extraction, we added 50 g of the limestone samples to about 125 mL sterile water, washed them with an ultrasonic cleaner for 15 s to ensure that the microorganisms on the limestone were washed into the sterile water, and then passed the solution through a 0.02-μm filter membrane. Next, 50 g of the limestone samples was used for pH determination and 50 g was converted into powder with a ball mill and passed through a 0.053-mm sieve for X-ray diffraction (XRD) analysis (to investigate the structure of the limestone samples) and Fourier transform infrared spectroscopy (FTIR) analysis (to characterize the atomic groups in the limestone samples). The remainder of the limestone samples were passed through a 0.053-mm sieve to obtain the powder remaining on the rock surface, which was placed in plastic bags for physicochemical experiments. We assessed the organic nitrogen (ON) and total carbon (TC) content of the powder samples using an organic elemental analyzer (all the C obtained by the analyzer should represent the TC because most of the samples are carbonate rocks). In addition, we assessed the organic carbon (OC) content of the powder samples using the H_2_SO_4_-K_2_Cr_2_O_7_ heating method (Bao, 2000).

### 2.3. DNA extraction and PCR amplification

Total microbial genomic DNA was extracted from the membrane (0.02-μm) samples using an E.Z.N.A.® soil DNA Kit (Omega Bio-tek, Norcross, GA, United States) according to the manufacturer’s instructions. The quality and concentration of DNA were determined using 1.0% agarose gel electrophoresis and a NanoDrop® ND-2000 spectrophotometer (Thermo Scientific Inc., United States). The DNA was then kept at −80°C prior to further use.

For bacteria, the V3–V4 hypervariable regions (468 bp) of the 16S rRNA gene were targeted using primer pairs 338F (5′-ACTCCTACGGGAGGCAGCAG-3′) and 806R (5′-GGACTACHVGGGTWTCTAAT-3′; Liu et al., 2016). For fungi, the internal transcribed spacer region (about 300 bp) was targeted using primer pairs ITS1F (5′-CTTGGTCATTTAGAGGAAGTAA-3′) and ITS2R (5′-GCTGCGTTCTTCATCGATGC-3′; Adams et al., 2013). The 16S PCR reaction mixture included 4 μL 5 × Fast Pfu buffer, 2 μL 2.5 mM dNTPs, 0.8 μL each primer (5 μM), 0.4 μL Fast Pfu polymerase, 10 ng template DNA, and ddH_2_O to give a final volume of 20 μL. The ITS PCR reaction mixture included 2 μL 10 × buffer, 2 μL 2.5 mM dNTPs, 0.8 μL each primer (5 μM), 0.2 μL rTaq polymerase, 0.2 μL bovine serum albumin, 10 ng template DNA, and ddH_2_O to give a final volume of 20 μL.

The PCR amplification cycling conditions were as follows: initial denaturation at 95°C for 3 min, denaturing at 95°C for 30 s (27 cycles for 16S and 35 cycles for ITS), annealing at 55°C for 30 s, and extension at 72°C for 45 s, and single extension at 72°C for 10 min, ending at 4°C. All samples were amplified in triplicate. The PCR products were extracted after 2% agarose gel electrophoresis and purified using an AxyPrep DNA Gel Extraction Kit (Axygen Biosciences, Union City, CA, United States) according to the manufacturer’s instructions. They were then quantified using a Quantus™ Fluorometer (Promega, United States). Purified amplicons were pooled in equimolar amounts and paired-end sequenced on an Illumina MiSeq PE300 platform (Illumina, San Diego, United States) according to standard protocols by Majorbio Bio-Pharm Technology Co. Ltd. (Shanghai, China). The raw sequencing reads were deposited into the US National Center for Biotechnology Information (NCBI) Sequence Read Archive (SRA) database (accession number: PRJNA944278).

### 2.4. Sequencing data processing and quality control

Raw FASTQ files were de-multiplexed using an in-house perl script, quality-filtered using fastp v0.19.6 (Chen et al., 2018), and merged using FLASH v1.2.7 (Magoč and Salzberg, 2011) based on the following criteria: (i) 300-bp reads were truncated at any site with a mean quality score of <20 over a 50 bp sliding window and truncated reads <50 bp were discarded, (ii) reads containing ambiguous characters were also discarded, and (iii) only overlapping sequences >10 bp were assembled according to their overlapping sequence. The maximum mismatch ratio of the overlapping region was set at 0.2. Reads that could not be assembled were discarded. The optimized sequences were then clustered into operational taxonomic units (OTUs) using UPARSE v7.1 (Edgar, 2013) with a 97% sequence similarity level. The most abundant sequence for each OTU was selected as a representative sequence. The taxonomy of each OTU representative sequence was analyzed using RDP Classifier v2.2 (Wang et al., 2007) and 16S and ITS rRNA gene databases (Silva v138 for bacteria and Unite v8.0 for fungi) using a confidence threshold of 0.7.

All sequences classified as chloroplast or mitochondria sequences were removed using the Majorbio Cloud platform (https://cloud.majorbio.com; Ren et al., 2022). Next, we selected the OTUs that were detected in ≥2 samples and that accounted for ≥5 occurrences across samples. Thereafter, samples were rarefied to the smallest observed number of reads to normalize for uneven sequencing effort.

### 2.5. Statistical analyses

All analyses were performed in the R Environment v4.2.2, and all plots were generated using the *ggplot2* package. The sequencing data were transformed to proportions using total-sum scaling (TSS) normalization (McKnight et al., 2019); the data were transformed using log10(*x* + *x*_0_), where *x* is the original non-zero abundance count data and *x*_0_ = 0.1·min(*x*) (Sunagawa et al., 2015). We used the pcoa() function in the *ape* package for unconstrained principal coordinate analysis (PCoA; Paradis et al., 2004). Permutational multivariate ANOVA (PerMANOVA) was performed with the adonis() function implemented in the *vegan* package (Dixon, 2003). We calculated the difference in richness between treatment groups using the aov() function in the *stats* package and the duncan.test() function in the *agricolae* package to perform a *post hoc* test (Steel and Torrie, 1980). We performed a two-sample permutation Student’s *t*-test (one-tailed; Hervé, 2022) using the perm.t.test() function in the *RVAideMemoire* package.

To construct Oa, Ia, Ia + Nut, and Nut co-occurrence networks, we selected OTUs that were present in ≥8 of all 15 Ia, Oa, Nut, or Ia + Nut samples (each treatment group had five concentration subgroups and three replicates). We then calculated the correlation coefficient *R* and *p value* between pairs of OTUs using the corAndPvalue() function in the *WGCNA* package (Langfelder and Horvath, 2012), and we identified eligible pairs based on absolute *R* > 0.75 and *value of p* < 0.01. The *igraph* package (Csardi and Nepusz, 2006) was used for network construction. Regarding the network topology properties, we measured the relative importance of a network node in terms of the information centrality of the node, and used the ratio between the reduced value of the network efficiency after removing any node and the network efficiency of the network without removing any node as the information centrality of that arbitrary node, and we used the information centrality of the largest node in the network as the network vulnerability indicator (Shang et al., 2021). We used the glmer() function in the *lme4* package to fit a generalized linear mixed-effect model (GLMM; Bates et al., 2015), with different treatments as random effects. The glmm.hp.() function in the *glmm.hp* package was used to calculate the relative contribution of multiple environmental factors after performing GLMM based on hierarchical partitioning theory (Lai et al., 2022).

## 3. Results

### 3.1. Rock properties and climatic conditions

The XRD results after standard mapping comparison indicate that the main phase of our rock samples was carbonatite (Reig et al., 2002; Zhang et al., 2017; Supplementary Figure S2A). In addition, the strong absorption peak at point b in the FTIR map was at around 1,419 cm, which represents the stretching vibration within [CO_3_]^2−^, followed by point c at 875 cm and point d at 711 cm, which represent the bending vibration within [CO_3_]^2−^ (Reig et al., 2002; Shareef et al., 2008), while point a at 3,444 cm was produced by the water absorption of KBr during the production process (Yang et al., 2015; Supplementary Figure S2B). In summary, the XRD and FTIR results indicate that the main component of our sample was calcium carbonate.

Based on the meteorological data obtained from the *in situ* weather station we installed (Supplementary Figure S2C), the mean temperature at our test site during the 60-day period (from 2022-01-15 to 2022-03-15) was 4.56°C (−4.7 to 25.5°C), the mean relative humidity was 82.44% (18.30–99.90%), the accumulated rainfall was 85.6 mm, and the mean atmospheric pressure was 89 kPa (87.8–90 kPa).

### 3.2. Bacterial and fungal community diversity among treatments

Among the 66 samples (3 RS, 3 CK, 15 Ia, 15 Oa, 15 Nut, and 15 Ia + Nut samples), 7,417 distinct fungal OTUs were obtained from 4,020,249 high-quality sequences and 2,754 distinct bacterial OTUs were obtained from 2,665,071 high-quality sequences at a 97% similarity level. After retaining the eligible OTUs (detected in ≥2 samples and accounting for ≥5 occurrences across samples), there were 2,832 fungal OTUs and 529 bacterial OTUs. The sequence count data were normalized based on the minimum value. The diversity indices were then calculated based on these data. The Good’s Coverage of the 66 samples varied from 99.43 to 99.80% for bacterial communities, with a mean of 99.65%, and from 98.78 to 99.97% for fungal communities, with a mean of 99.42% (Supplementary Table S2). The dilution curves (Supplementary Figure S3), theoretical species richness [Chao1 and abundance-based coverage estimator (ACE)], and Good’s Coverage showed that after 60 days, a certain number of microorganisms had colonized the surface of the limestone sand-sized grains, and the diversity of the fungal communities was higher than that of the bacterial communities.

**Figure 1:**
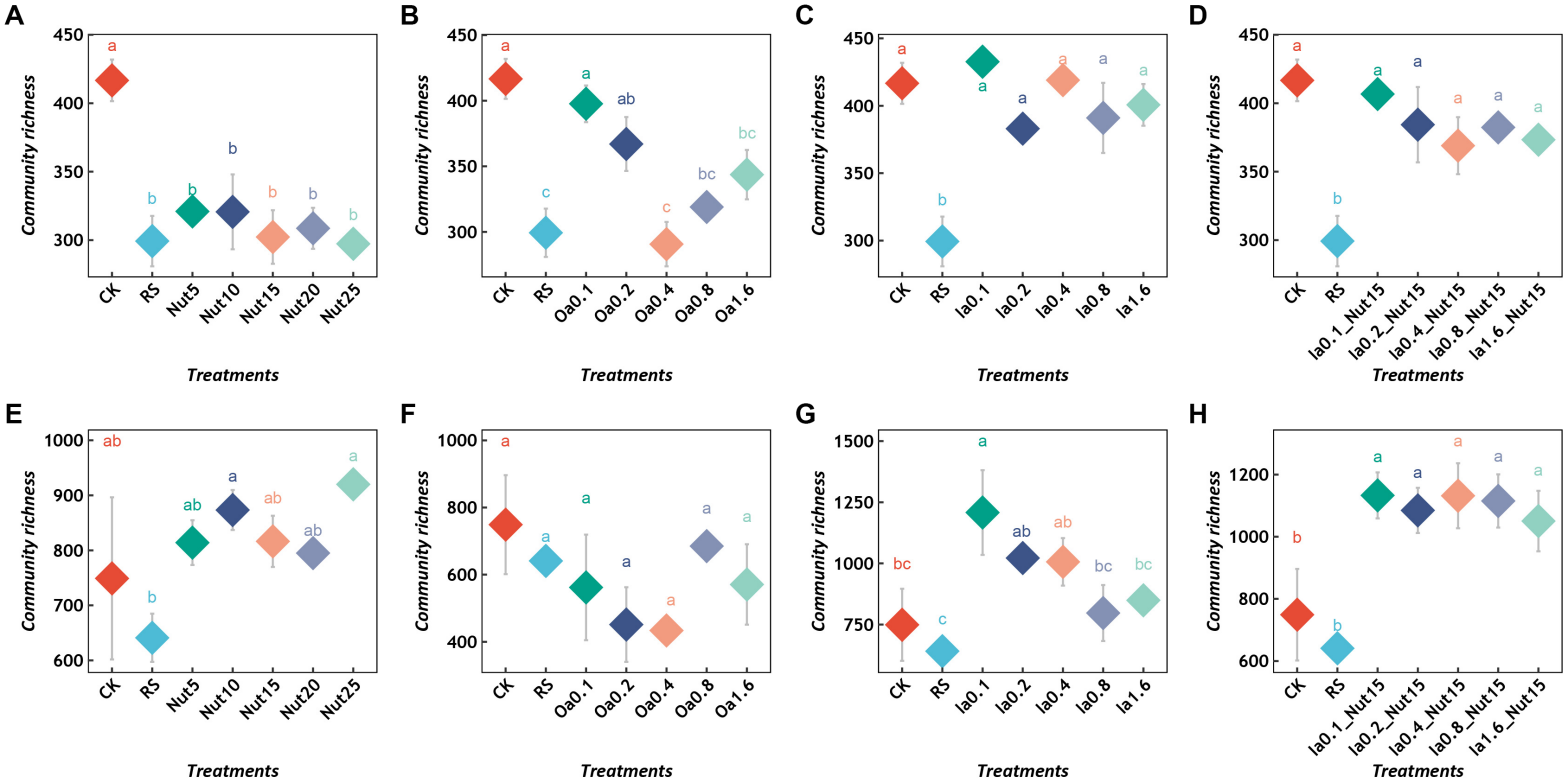
Richness of bacterial **(A–D)** and fungal **(E–H)** communities under different treatment conditions. Different lowercase letters indicate significant differences between groups (*p* < 0.05), and the same lowercase letters indicate no significant differences between groups. RS, rain shade; Nut, nutrient; Oa, organic acid; Ia, inorganic acid; la + Nut, inorganic acid + Nutrient; and values after each treatment indicate concentration.

Regarding the bacterial communities, all Nut concentrations and high Oa concentrations significantly reduced the richness (Figures 1A,B), while the Ia and Ia + Nut treatments did not significantly alter the richness (Figures 1C,D). In addition, RS treatment significantly reduced bacterial richness (Figures 1A–D). Regarding the fungal communities, RS treatment did not significantly change the richness (Figures 1E–H). In addition, compared to CK, all Ia + Nut treatments and low Ia concentrations significantly increased the fungal richness (Figures 1G,H), while the Nut and Oa treatments did not significantly increase the richness (Figures 1E,F).

Rain shade treatment did not significantly change the evenness of the bacterial or fungal communities (Supplementary Figures S4A–H). High Ia + Nut treatment significantly reduced the bacterial community evenness (Supplementary Figures S4C,D), while the other treatments did not significantly change it. In addition, the Nut treatments changed the fungal community evenness (Supplementary Figure S4E), while the other treatments did not.

### 3.3. Comparison of bacterial and fungal community composition among treatments

**Figure 2:**
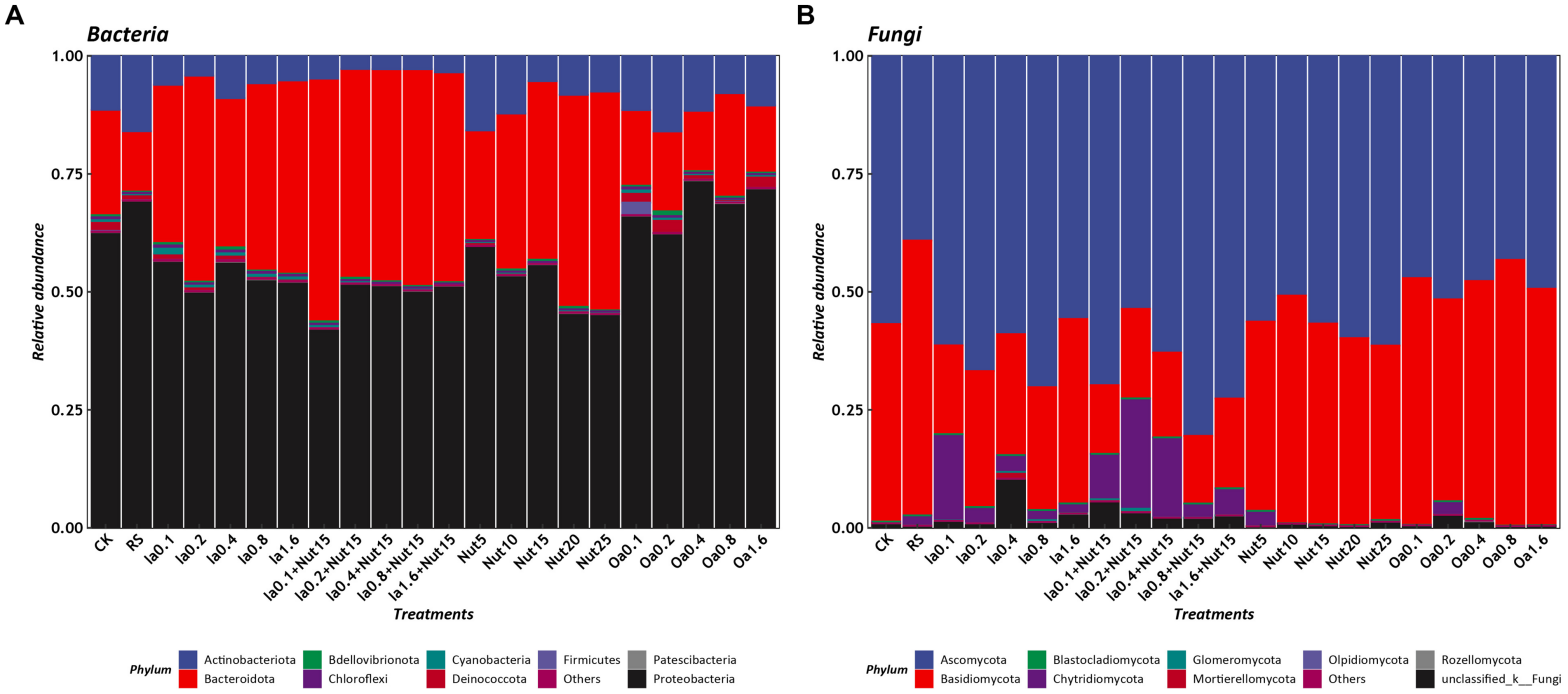
Relative abundances of phyla under different treatment conditions (**A**: bacteria, **B**: Fungi). RS, rain shade; Nut, nutrient; Oa, organic acid; Ia, inorganic acid; Ia + Nut, inorganic acid + nutrient; values after each treatment indicate concentration.

The dominant bacterial phyla based on mean relative abundance (>1% threshold) among all treatments were Proteobacteria (56.78%), Bacteroidota (32.85%), and Actinobacteriota (8.22%). In contrast, the mean relative abundances of Deinococcota (0.95%), Cyanobacteria (0.42%), Bdellovibrionota (0.40%), Firmicutes (0.15%), Chloroflexi (0.11%), and Patescibacteria (0.05) were < 1% (Figure 2A). The top 10 bacterial genera were *Flavobacterium* (22.34%), *Massilia* (19.19%), *Noviherbaspirillum* (6.95%), *Cytophaga* (7.41%), *Cellvibrio* (5.34%) *Caulobacter* (5.46%), *Arthrobacter* (5.27%), *Pseudomonas* (4.34%), and *Brevundimonas* (2.68%; Supplementary Figure S5A).

The dominant fungal phyla based on mean relative abundance (>1% threshold) among all treatments were Ascomycota (57.82%), Basidiomycota (35.58%), and Chytridiomycota (4.45%). In contrast, the mean relative abundance of Mortierellomycota (0.15%) was <1% (Figure 2B). The top six fungal genera were *Epicoccum* (12.67%), *Symmetrospora* (9.37%), *Cladosporium* (9.05%), *Vishniacozyma* (4.62%), *Itersonilia* (4.47%), and *Botrytis* (2.19%; Supplementary Figure S5B).

### 3.4. Differences in microbial communities among treatments

**Figure 3:**
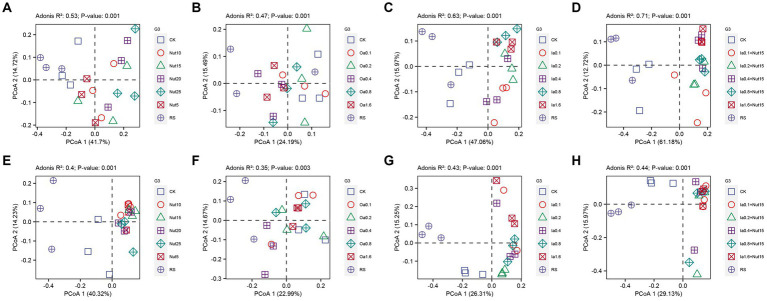
Principal coordinate analysis (PCoA) of bacterial **(A–D)** and fungal **(E–H)** communities under different treatment conditions. RS, rain shade; Nut, nutrient; Oa, organic acid; Ia, inorganic acid; Ia + Nut, inorganic acid + nutrient; and values after each treatment indicate concentration.

Our PCoA and Adonis tests showed that bacterial and fungal communities exhibited significant separation regarding different Nut, Oa, Ia, and Ia + Nut concentrations, and that bacterial communities (0.71 > *R*^2^ > 0.47, *p* = 0.001) differed more than fungal communities (0.44 > *R*^2^ > 0.35, *p* = 0.001) for the treatments (Figure 3). In addition, UpSet plots showed that most OTUs were shared between the different treatments (Supplementary Figure S6).

We calculated the Bray–Curtis dissimilarity distances for bacterial and fungal communities between the treatment groups and the CK group, and the treatment groups and the RS group. For bacteria, there was a significant Nut effect, with the distances significantly increasing with Nut concentration (Supplementary Table S3; Supplementary Figures S7A,B). However, there were no significant acidity (Oa, Ia, or Ia + Nut) effects (Supplementary Figure S7). For fungi, there were no significant differences in the Bray–Curtis dissimilarity distance among the treatment groups and CK, or the treatment groups and RS (Supplementary Figure S8).

In summary, there were significant differences in bacterial and fungal community composition among the groups, but a large proportion was shared. In addition, the bacterial communities were more sensitive to the treatments than the fungal communities, especially regarding Nut concentrations.

### 3.5. Bacterial and fungal co-occurrence networks

For both fungal and bacterial communities, the proportion of positive correlations (among all correlations) was higher than the proportion of negative correlations in the Oa, Ia, Ia + Nut, and Nut networks, while the Nut network had a greater proportion of negative correlations. More negative correlations indicate increased competition between species, which occurred for both fungal and bacterial communities in the Nut treatment group (Supplementary Figure S9; Supplementary Table S4).

**Figure 4:**
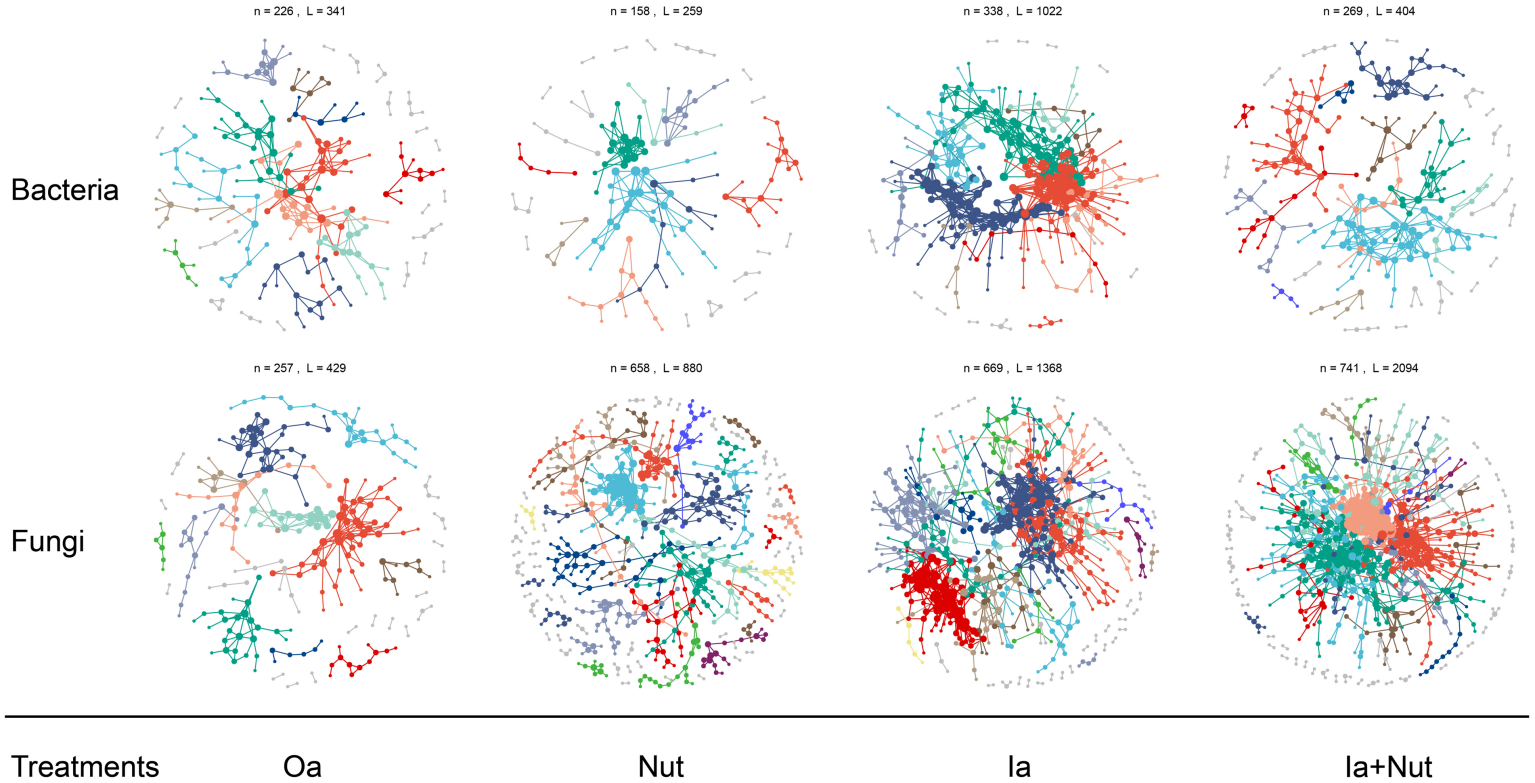
Bacterial and fungal co-occurrence networks on limestone under different treatment conditions. Large modules with ≥5 nodes are shown in different colors, and smaller modules are shown in gray. RS, rain shade; Nut, nutrient; Oa, organic acid; Ia, inorganic acid; Ia + Nut, inorganic acid + nutrient.

For the Nut network, strong correlations (*R* > 0.25) were more common among bacterial OTUs than fungal OTUs, while weak correlations (−0.25 < *R* < 0.25) were less common among bacterial OTUs than fungal OTUs. For the Oa and Ia + Nut networks, the negative correlations were stronger among bacterial OTUs than fungal OTUs, while the positive correlations were weaker among bacterial OTUs than fungal OTUs. For the Ia network, the negative correlations were weaker among bacterial OTUs than fungal OTUs, while the positive correlations were stronger among bacterial OTUs than fungal OTUs (Figure 4; Supplementary Figure S9).

When considering the significant correlations for a given threshold (*R* > 0.75, *p* < 0.01), the number of positive correlation edges was greater than the number of negative correlation edges for both fungal and bacterial communities (Supplementary Table S4). In addition, the permutation Student’s *t*-test showed that the number of nodes was significantly lower in the bacterial networks than the fungal networks, but the edge density (i.e., the ratio of the number of edges to the number of all possible edges) was significantly higher (Supplementary Table S5). A higher edge density indicates more efficient network information transfer, higher resilience to environmental stress, and easier achievement of dynamic stability. The networks showed that the bacterial communities were more tightly connected, more complex, and more resilient to environmental stress, but less resistant to environmental stress than the fungal communities.

### 3.6. Relative contribution of environmental factors to microbial richness

**Figure 5:**
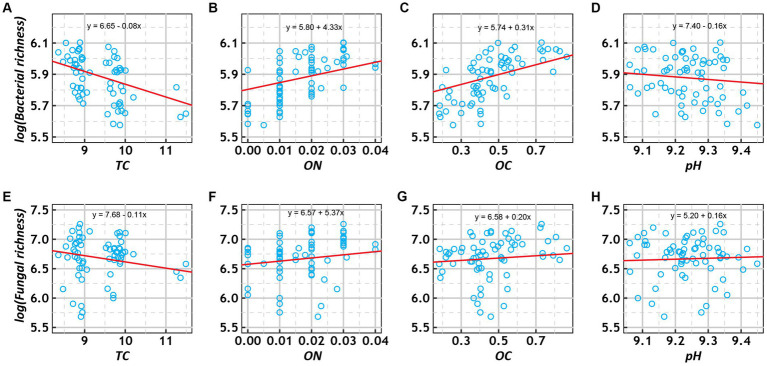
Scatter plots of the relationships of log-transformed richness (bacterial: **A–E**; fungal: **E–H**) with total carbon (TC, %), organic nitrogen (ON, %), organic carbon (OC, %), and pH under various treatment conditions. The trend lines represent the best-fit lines according to generalized linear mixed-effects models (GLMMs).

Generalized linear mixed-effect models showed that bacterial and fungal richness increased with OC and ON and decreased with TC among the various treatments (Figure 5). In addition, fungal richness increased with pH, while bacterial richness decreased with pH. To investigate the relative contributions of the above four environmental factors to bacterial and fungal richness, we calculated their contributions after multivariate GLMM modeling based on hierarchical partitioning (Supplementary Figure S10). The results showed that OC had the highest relative contribution to bacterial richness, while TC had the highest relative contribution to fungal richness.

## 4. Discussion

### 4.1. Bacterial and fungal communities on limestone

The bacterial taxa found in this study are similar to those found in previous studies (Miller et al., 2008, 2009; Miller, 2010; Chimienti et al., 2016), but differ in terms of the dominant taxa. At the phylum level, the dominant bacteria found in this study were Proteobacteria (56.78%), Bacteroidota (32.85%), and Actinobacteriota (8.22%; Figure 2A). Proteobacteria is a key chemolithotroph involved in biotic degradation (Scheerer et al., 2009; Miller, 2010), most taxa of Bacteroidota are halophiles, and Actinobacteriota can lower the pH of rock surfaces and can be used as an indicator of biodeterioration (Scheerer et al., 2009). The microbial communities on the surfaces of Italian and French limestone tombstones and monasteries were reported to be dominated by Cyanobacteria and Alphaproteobacteria (Mihajlovski et al., 2017; Gambino et al., 2021). The microbial communities on the surfaces of 149 limestone and granite gravestone samples from three continents were reported to be dominated by Proteobacteria, Cyanobacteria, and Bacteroidetes (Brewer and Fierer, 2018).

At the phylum level, the dominant fungi found in this study were Ascomycota (57.82%) and Basidiomycota (35.58%; Figure 2B), similar to results from a previous study (Gómez-Cornelio et al., 2012). At the genus level, some of the rock-inhabiting fungal species (belonging to the genera *Cladosporium*, *Epicoccum*, and *Botrytis*) were the same as those found in previous studies (Gómez-Cornelio et al., 2012; Trovão et al., 2020), while some endemic taxa (such as *Vishniacozyma*, *Itersonilia*, and *Symmetrospora*) were also found (Supplementary Figure S5B). It is worth noting that some species of the genera *Cladosporium*, *Epicoccum*, and *Botrytis* have been found to have a potential for biodeterioration (Gómez-Cornelio et al., 2012; Li et al., 2018; Trovão et al., 2020).

### 4.2. Response of bacterial and fungal communities to different treatments

Cyanobacteria are often the first colonizers and first microbes to perform ecosystem functions because their photoautotrophic metabolism is based on the ability to use light to produce energy and organic matter and to collect micronutrients, oxygen, carbon dioxide, and water from the surrounding air (Pinheiro et al., 2019). However, the relative abundance of Cyanobacteria in this study was low (Figure 2A). It is important to note that the above studies all assessed bacterial composition patterns under natural conditions, whereas in this study, Oa and Ia treatments were imposed (Supplementary Figure S1). This led to the interesting phenomenon of inorganic material produced by limestone dissolution allowing Proteobacteria, which depends on chemoenergetic inorganic nutrients, to colonize the limestone surfaces in large numbers (Figure 2A).

The dominant fungi differ between different climatic conditions, with filamentous fungi dominating in mild and humid environments, and the so-called microcolonial black fungi dominating in arid and semi-arid climates (Sterflinger and Piñar, 2013; Selbmann et al., 2015; Pinheiro et al., 2019). In our experimental site, which is located in a subtropical climate zone, filamentous fungi such as *Cladosporium* and *Epicoccum* were two of the dominant genera on the limestone surfaces (Supplementary Figure S5B), which is similar to what has been reported previously (Gómez-Cornelio et al., 2012; Pinheiro et al., 2019; Trovão et al., 2020).

Fungi can secrete more Oa than bacteria (Abdel Ghany et al., 2019) and grow mycelia that can damage rocks (Gaylarde et al., 2003; Dakal and Cameotra, 2012; Trovão et al., 2019). In this study, different concentrations of acids/nutrients shaped the bacterial and fungal communities; the bacterial communities were less resistant to the environmental stresses (different concentrations of acids/nutrients) than the fungal communities, so the abundance of fungi was higher than that of bacteria (Figure 3). Among the fungal genera found, *Cladosporium* and *Epicoccum* were both filamentous fungi; they may have been dominant because mycelia increase the efficiency of nutrient uptake (Fukasawa et al., 2020), which can increase the competitiveness of fungi compared to bacteria. Therefore, it is unsurprising that the abundance of fungi was higher than that of bacteria for the acid/nutrient treatments.

### 4.3. Adaptation of limestone lithogenic microorganisms

Typically, fungal communities are more able to persist in arid environments than bacterial communities and are more resistant to drought, while bacterial communities have good resilience under suitable conditions, i.e., they are able to recover rapidly (Barnard et al., 2013; de Vries et al., 2018). The limestone samples were in a chronic water deficit environment, which resulted in higher fungal abundance than bacterial abundance and a higher resistance to environment stress among the fungi compared to the bacteria, which were more resilient to the arid environment than the fungi (Figures 1, 3, 4; Supplementary Table S5). In addition, among the different treatments, Nut decreased bacterial richness (Figure 1A). Conversely, Ia + Nut increased fungal richness (Figure 1H). We hypothesized that fungi are better adapted to the limestone surface environment and are more competitive than bacteria during the early colonization process. The species richness and evenness of the fungal community were higher than those of the bacterial community after the sterile limestone sand-sized grains were subjected to natural conditions for 60 days (Figure 1; Supplementary Figure S4). Oa and Nut might hinder bacterial colonization, while Ia + Nut promoted fungal colonization, suggesting that the fungal community might be better adapted to limestone surfaces than bacteria (Figure 1). In addition, we speculate that rainfall may be the main source of bacterial communities on limestone surfaces, while the environment may be the main source of fungal communities (Figure 1).

Based on our findings, we believe that limestone can be protected from biodeterioration in several ways. First, biodeterioration is the result of a combination of physical and biochemical mechanisms (Gadd, 2017b). Among the biochemical mechanisms, inorganic and organic acids are important influences. As carbonic acid is a very common inorganic acid that is mainly formed when excessively high concentrations of CO_2_ in the air dissolve in water (Gadd, 2017b; Liu et al., 2020), CO_2_ concentrations should be monitored to prevent carbonic acid in rainwater from dissolving limestone. Second, phototrophs such as Cyanobacteria are thought to be an important group of organisms in rock biodeterioration that are able to use photosynthesis to assimilate CO_2_ into organic forms for subsequent colonizers (Sand and Bock, 1991; Crispim et al., 2006; Vázquez-Nion et al., 2018). Therefore, protecting limestone from direct sunlight as much as possible will slow the growth of lithobiontic Cyanobacteria and thus reduce the biodeterioration of the limestone. Third, rainfall, as an important environmental factor, is associated with a variety of biodeterioration processes, such as discoloration, distortions, blackening, and patina formation (Liu et al., 2020). Hence, limestone surfaces should be protected from rainfall to reduce colonization by microorganisms in the rainfall. Fourth, as it has been found that microorganisms cultured from the rinds of biodeteriorated rock surfaces can still cause damage to rocks (Gómez-Cornelio et al., 2012), microorganisms growing on limestone rocks undergoing biodeterioration need to be removed to avoid further erosion.

## 5. Conclusion

This study investigated microbial colonization of limestone surfaces after 60 days of treatment with various Nut, Oa, Ia, Ia + Nut, and Nut concentrations, providing a new perspective on microbial–rock interactions. We draw the following three main conclusions. First, fungi and bacteria exhibited different colonization patterns during the 60 days that the limestone was left in its natural environment. Fungi were more resistant to environmental stress and able to colonize the limestone surfaces rapidly and in large numbers, showing higher richness and competitiveness, while bacterial communities, although less diverse, were more complex and resilient to environmental stress, with an increased ability to recover rapidly. Second, the rock surface environment (acid erosion and nutrient availability) determined the early colonization by microorganisms, with different concentrations of Nut, Oa, and Ia all shaping the microbial communities in different ways. The higher the acidity (Ia and Oa), the greater the differences (compared to the CK) in microbial communities. The bacteria were less resistant to environmental stress than the fungi, and there was an obvious Nut concentration gradient effect for bacteria. Third, the richness of bacterial and fungal communities were influenced by OC, ON, TC, and pH, with OC being an important determinant of bacterial community richness and TC being an important determinant of fungal community richness. However, this study lacks a quantitative analysis of how limestone surface characteristics such as roughness and porosity affect microbial colonization. In addition, the limestone surface environment changed drastically over time, and this study did not assess the microbial colonization in different periods. Therefore, studies on the ecological succession of microorganisms on limestone surfaces in different periods should be conducted to provide a scientific basis for the conservation of limestone artifacts and early ecological succession in karst areas.

## Data availability statement

The datasets presented in this study can be found in online repositories. The names of the repository/repositories and accession number(s) can be found at: https://www.ncbi.nlm.nih.gov/, PRJNA944278.

## Author contributions

LYu conceived the project. JC, FL, XZ, QZ, LZ, JL, and LYa collected samples in the field. JC, FL, XZ, YW, and LYan performed data analysis. JC, FL, and XZ performed the experiment. JC and LYu wrote the manuscript. All authors contributed to the article and approved the submitted version.

## Funding

This work was supported by the 13th Five-year National Key Research and Development Plan (grant number 2016YFC0502604); the Construction Program of Biology First-class Discipline in Guizhou (grant number GNYL[2017]009); and the Postgraduate Education Innovation Program in Guizhou Province (grant number YJSKYJJ[2021]079).

## Conflict of interest

The authors declare that the research was conducted in the absence of any commercial or financial relationships that could be construed as a potential conflict of interest.

## Publisher’s note

All claims expressed in this article are solely those of the authors and do not necessarily represent those of their affiliated organizations, or those of the publisher, the editors and the reviewers. Any product that may be evaluated in this article, or claim that may be made by its manufacturer, is not guaranteed or endorsed by the publisher.
