# Supplementary figures and images for: Nutrient availability and acid erosion determine the early colonization of limestone by lithobiontic microorganisms
Source: Front Microbiol. 2023 Jun 9;14:1194871. doi: 10.3389/fmicb.2023.1194871 (PMC10289080; doi:10.3389/fmicb.2023.1194871)

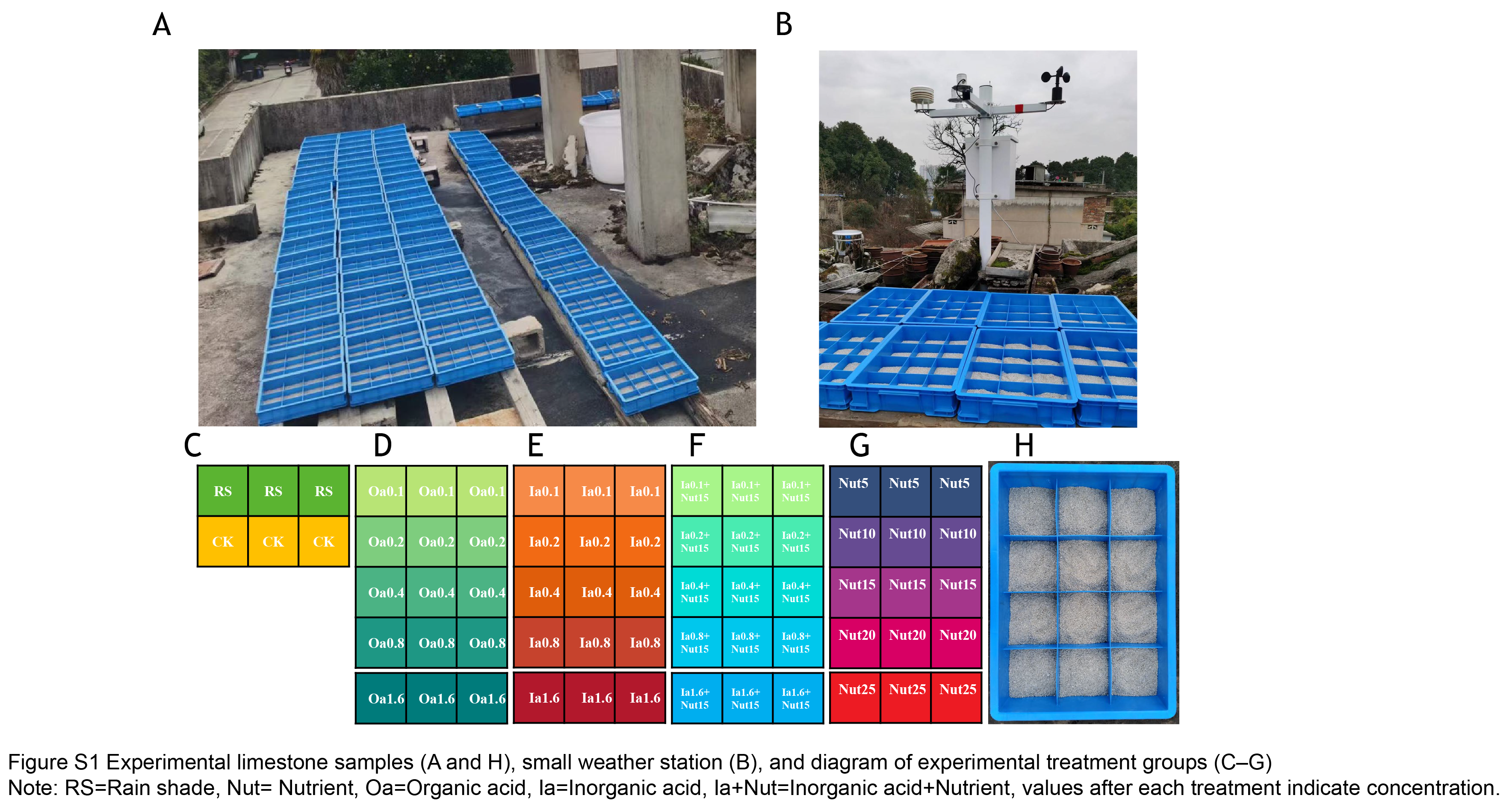

Supplement: Supplementary file 2 [file Image_1.PNG]

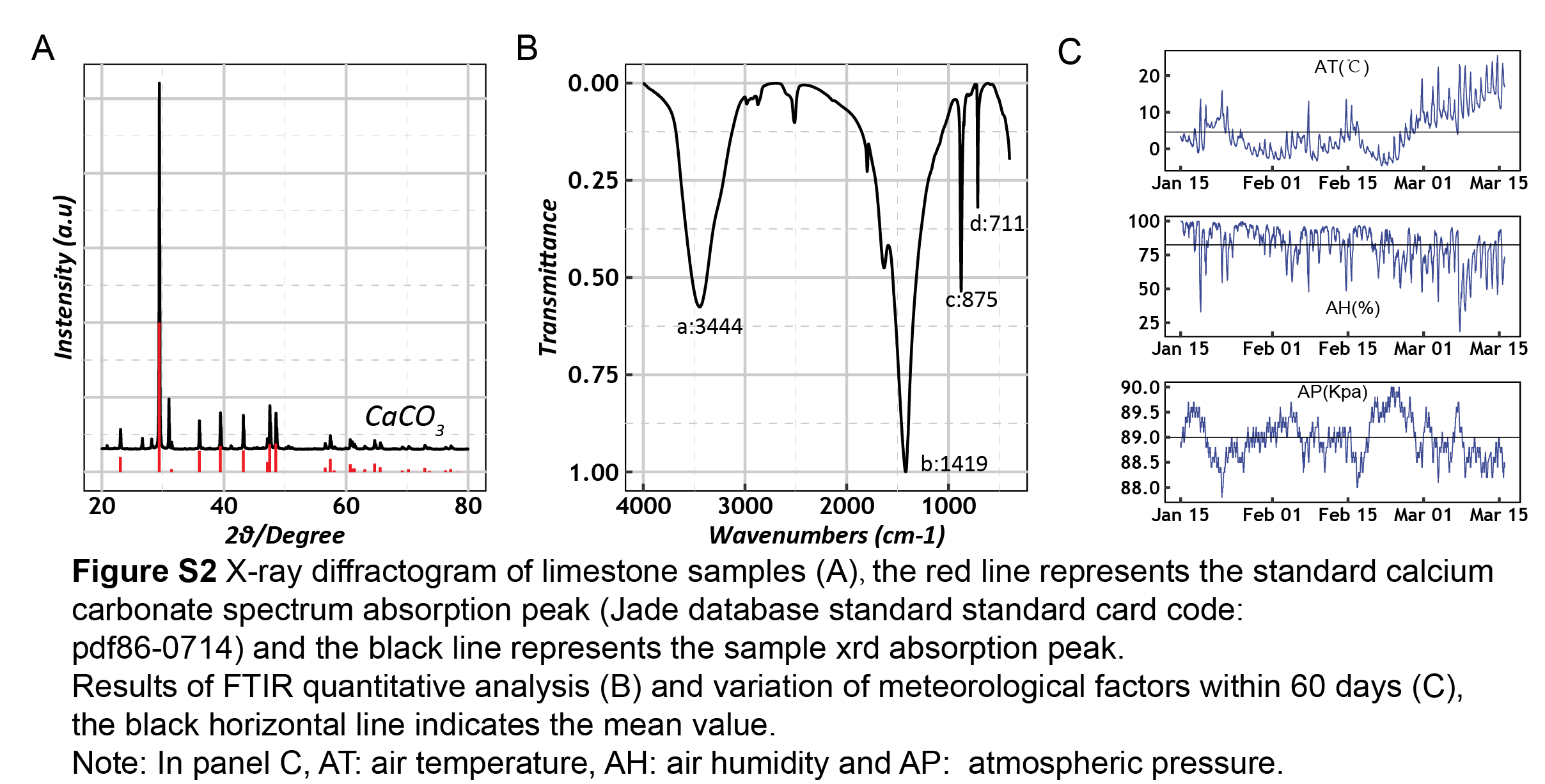

Supplement: Supplementary file 3 [file Image_2.PNG]

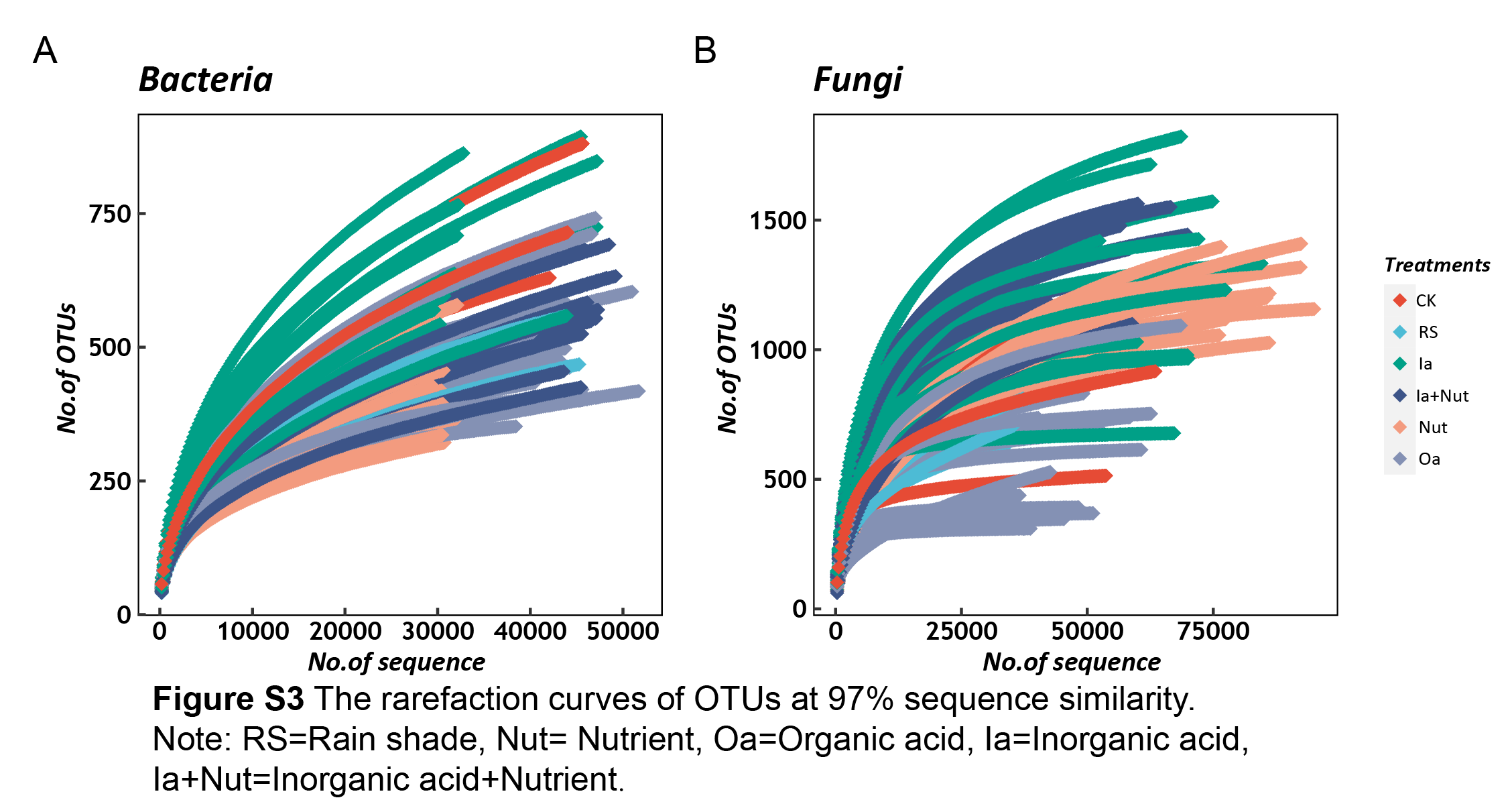

Supplement: Supplementary file 4 [file Image_3.PNG]

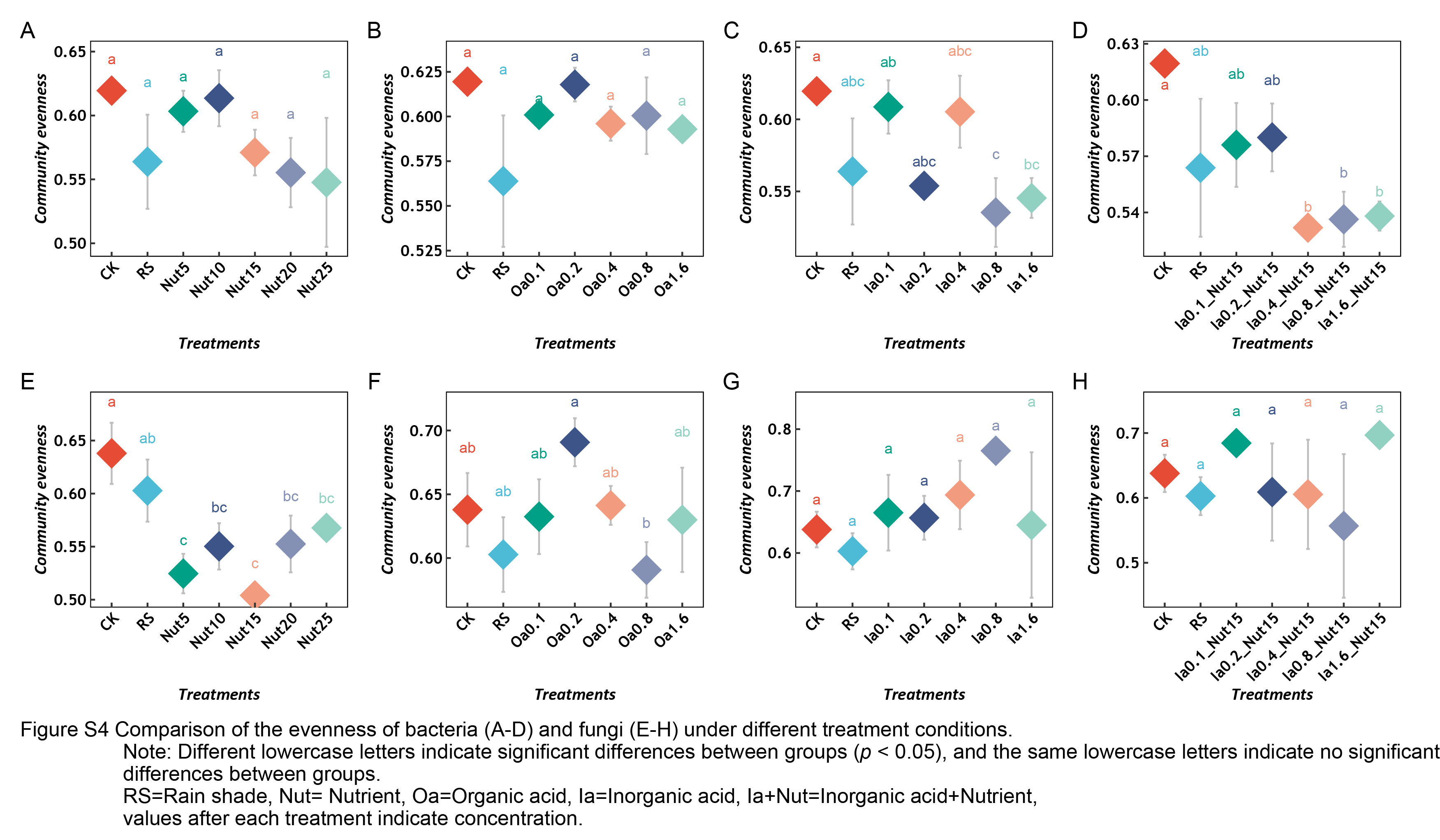

Supplement: Supplementary file 5 [file Image_4.PNG]

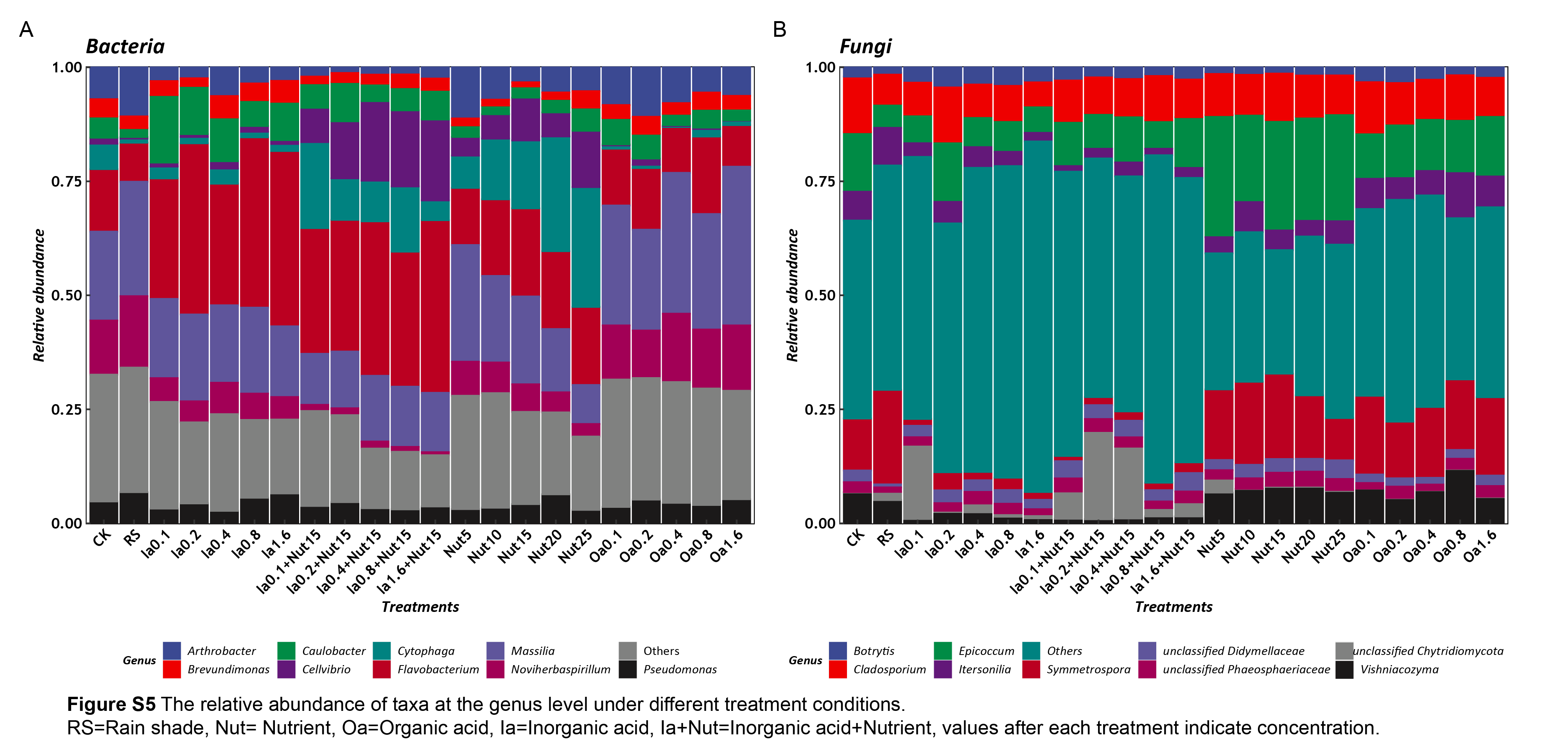

Supplement: Supplementary file 6 [file Image_5.PNG]

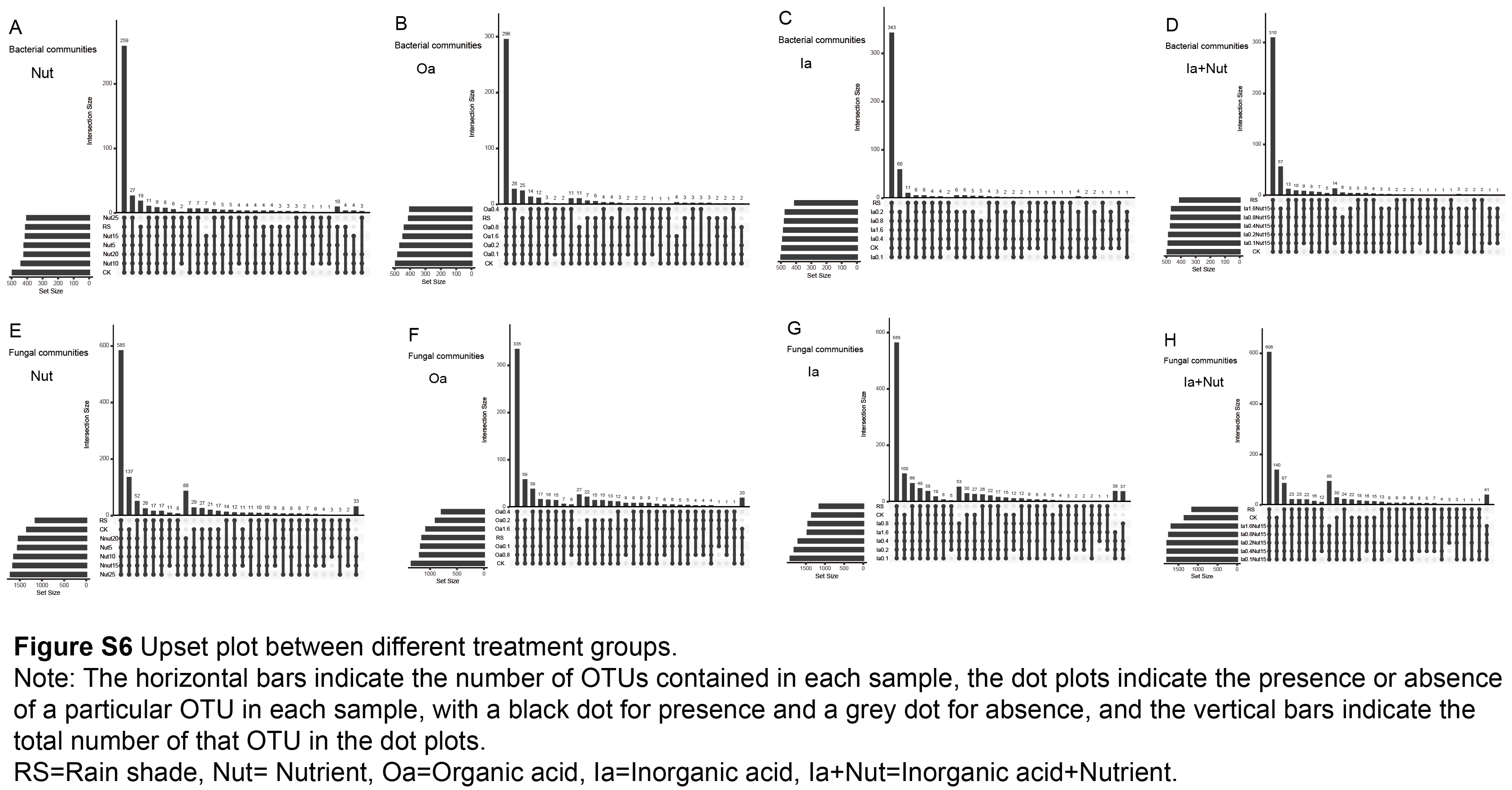

Supplement: Supplementary file 7 [file Image_6.PNG]

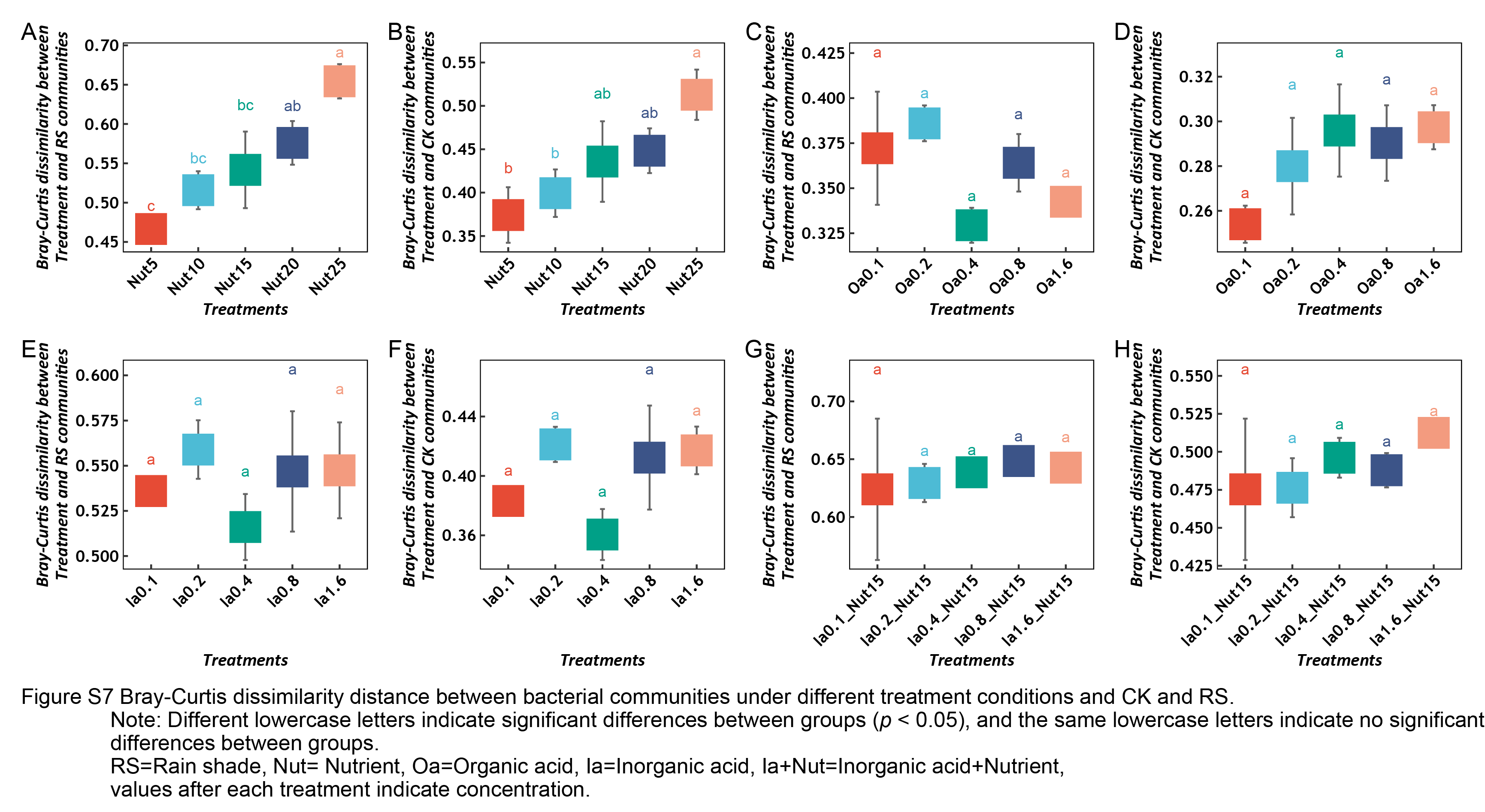

Supplement: Supplementary file 8 [file Image_7.PNG]

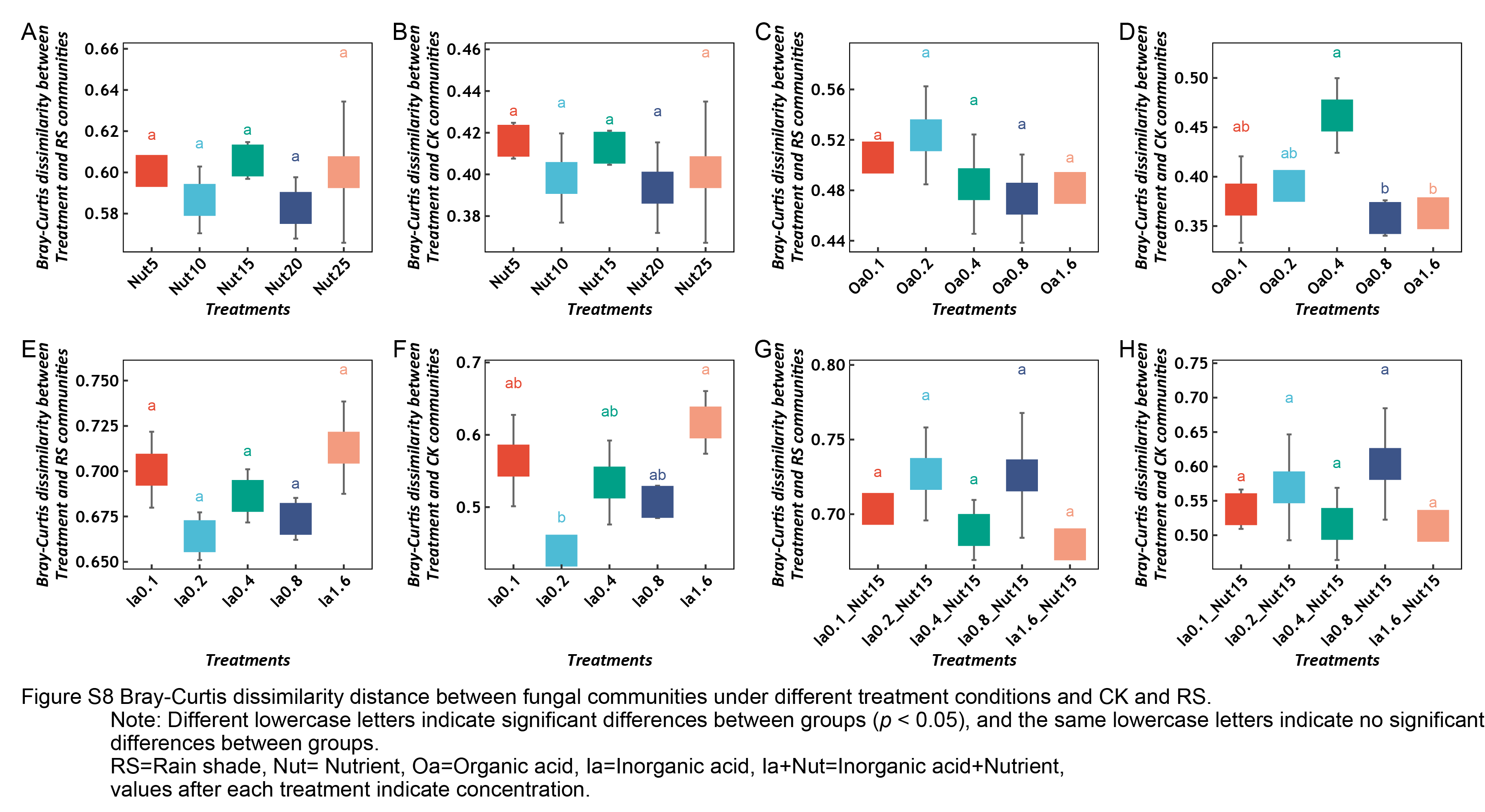

Supplement: Supplementary file 9 [file Image_8.PNG]

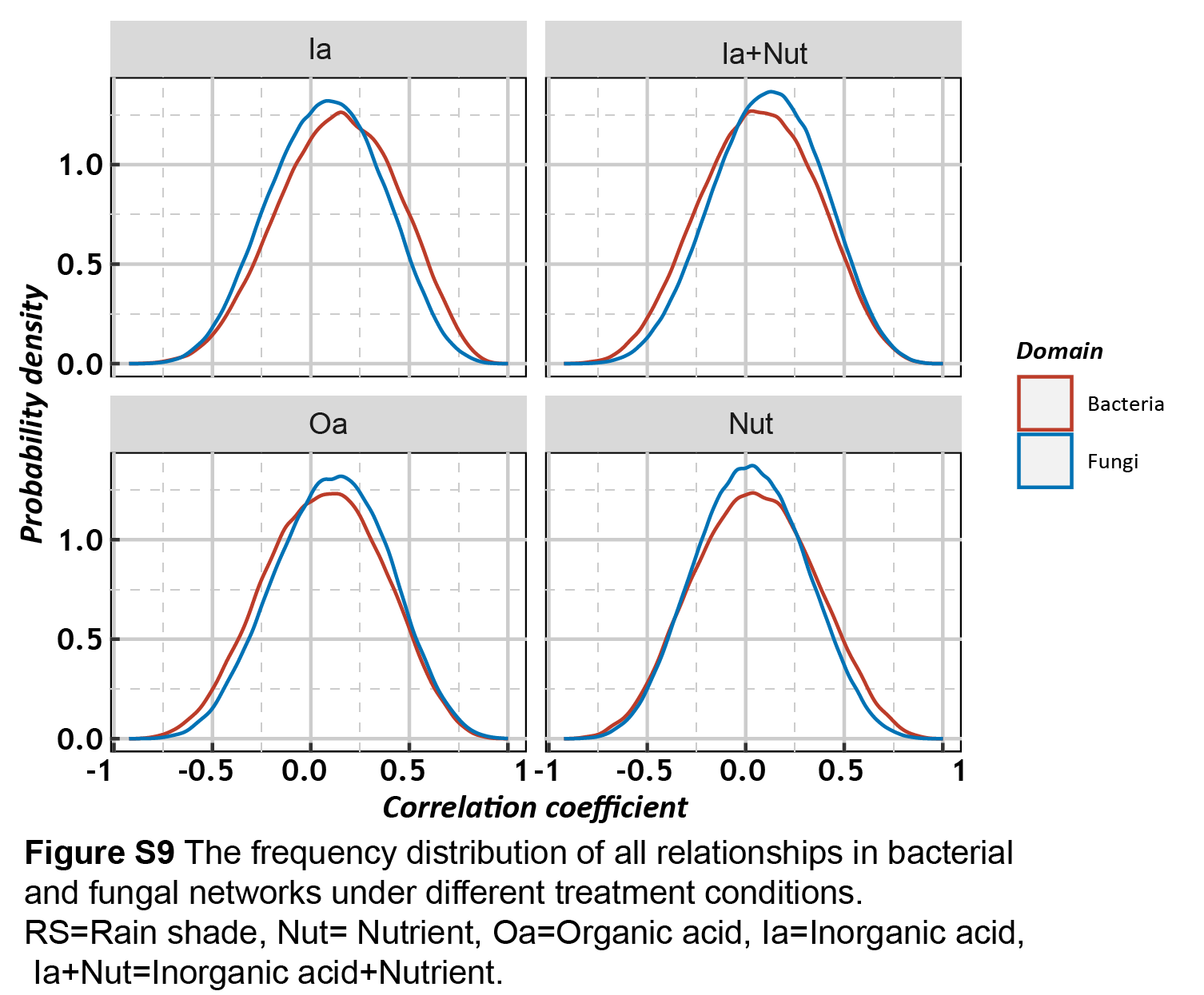

Supplement: Supplementary file 10 [file Image_9.PNG]

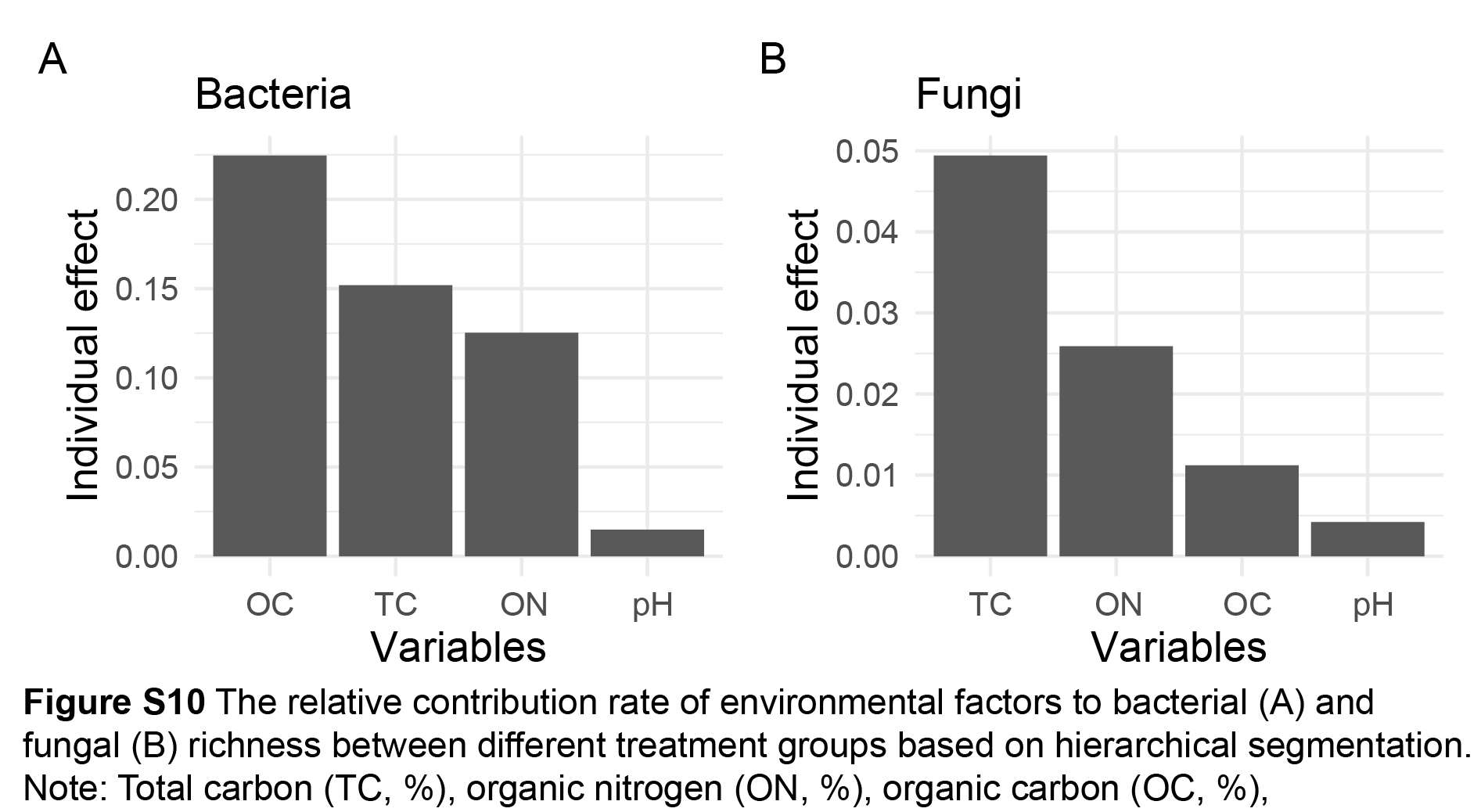

Supplement: Supplementary file 11 [file Image_10.PNG]
